# Supplementary figures and images for: Optimal Cannabinoid-Terpene Combination Ratios Suppress Mutagenicity of Gastric Reflux in Normal and Metaplastic Esophageal Cells
Source: bioRxiv. 2025 Sep 25:2025.09.23.678062. Preprint. [Version 1] doi: 10.1101/2025.09.23.678062 (PMC12485961; doi:10.1101/2025.09.23.678062)

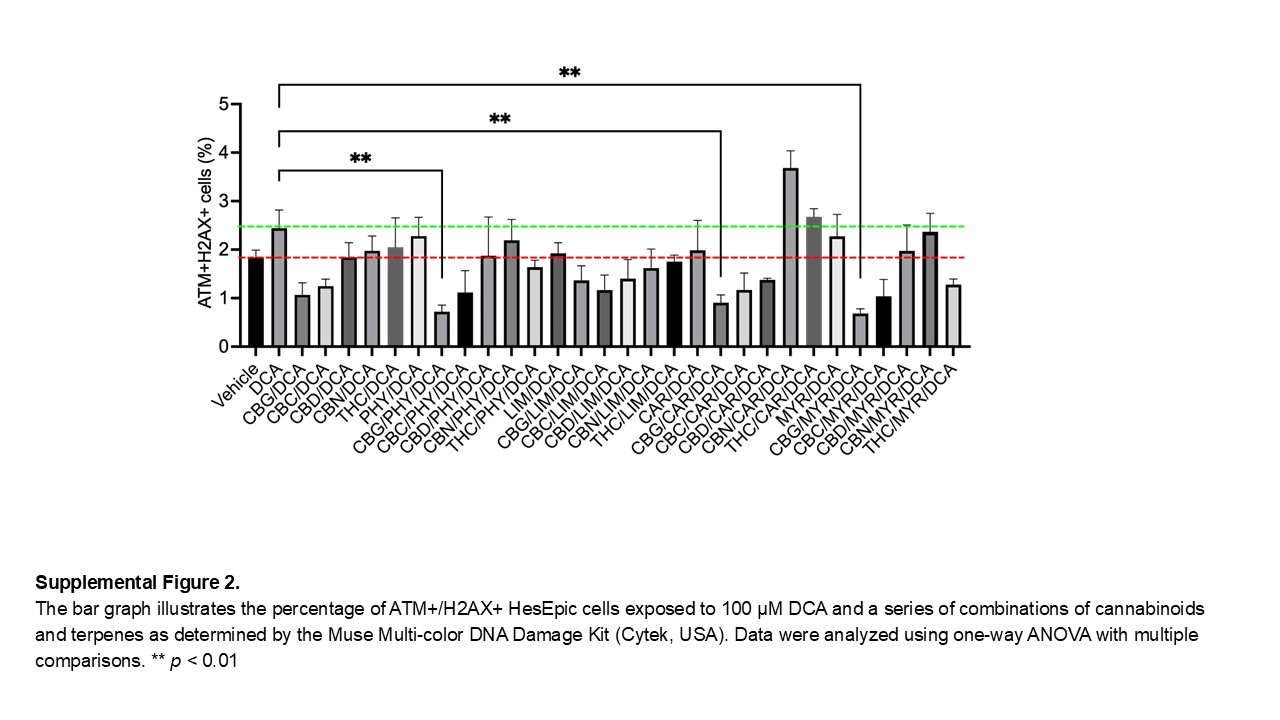

Supplement: Supplement 1 [file media-1.jpg]

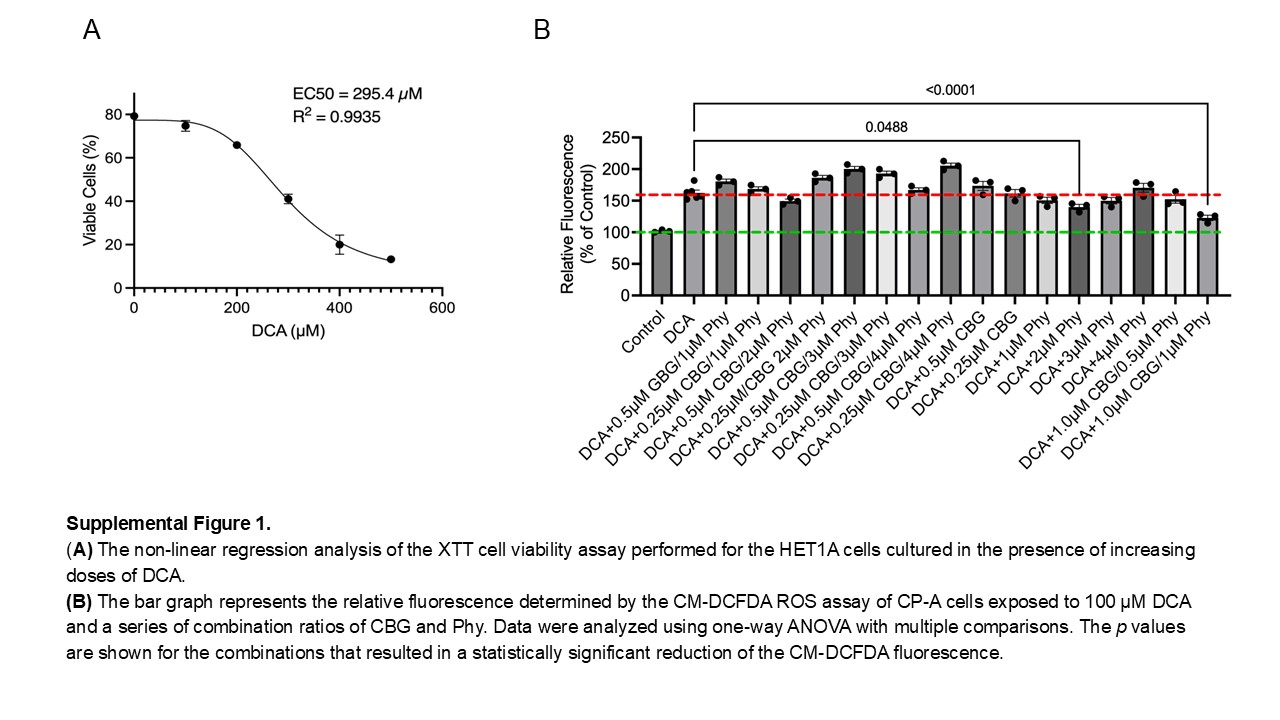

Supplement: Supplement 2 [file media-2.jpg]
